# Lestaurtinib inhibits Citron kinase activity and medulloblastoma growth through induction of DNA damage, apoptosis and cytokinesis failure

**DOI:** 10.3389/fonc.2023.1202585

**Published:** 2023-06-19

**Authors:** Gianmarco Pallavicini, Giorgia Iegiani, Roberta Parolisi, Alessia Ferraro, Francesca Garello, Valeria Bitonto, Enzo Terreno, Marta Gai, Ferdinando Di Cunto

**Affiliations:** ^1^ Neuroscience Institute Cavalieri Ottolenghi, Turin, Italy; ^2^ Department of Neuroscience ‘Rita Levi Montalcini’, University of Turin, Turin, Italy; ^3^ Department of Molecular Biotechnology and Health Sciences, University of Turin, Turin, Italy

**Keywords:** Lestaurtinib, CitK, drug repurposing, medulloblastoma, preclinical studies

## Abstract

**Introduction:**

Medulloblastoma (MB), the most common malignant pediatric brain tumor, is currently treated with surgery followed by radiation and chemotherapy, which is accompanied by severe side effects, raising the need for innovative therapies. Disruption of the microcephaly-related gene Citron kinase (CITK) impairs the expansion of xenograft models as well as spontaneous MB arising in transgenic mice. No specific CITK inhibitors are available.

**Methods:**

Lestaurtinib, a Staurosporine derivative also known as CEP-701, inhibits CITK with IC50 of 90 nM. We therefore tested the biological effects of this molecule on different MB cell lines, as well as in vivo, injecting the drug in MBs arising in SmoA1 transgenic mice.

**Results:**

Similar to CITK knockdown, treatment of MB cells with 100 nM Lestaurtinib reduces phospho-INCENP levels at the midbody and leads to late cytokinesis failure. Moreover, Lestaurtinib impairs cell proliferation through CITK-sensitive mechanisms. These phenotypes are accompanied by accumulation of DNA double strand breaks, cell cycle block and TP53 superfamily activation in vitro and in vivo. Lestaurtinib treatment reduces tumor growth and increases mice survival.

**Discussion:**

Our data indicate that Lestaurtinib produces in MB cells poly-pharmacological effects extending beyond the inhibition of its validated targets, supporting the possibility of repositioning this drug for MB treatment.

## 1 Introduction

Medulloblastoma (MB) is the most common malignant pediatric brain tumor (1, 2) and has been traditionally classified into four biological subgroups: WNT, Sonic Hedgehog (SHH), Group 3 and Group 4 (3–6). Due to the overlap between Group 3 and Group 4, it has recently been re-classified in: WNT-activated, SHH-activated and TP53-wildtype, SHH-activated and TP53-mutant, non-WNT/non-SHH (7). In general, MB is treated with surgery, followed by radiation of the entire neuro-axis and high dose multi-agent chemotherapy (2). Despite long-term survival can be as high as 90% in the rare WNT subgroup, it is on average around 50% in the other subtypes, with worse prognosis in non-WNT/non-SHH patients (8). Moreover, radio-chemotherapy is frequently accompanied by severe neurological, cognitive, and endocrine side effects (8). For these reasons, effective therapies less disruptive of normal physiology are an unmet medical need.

MB cells share many molecular pathways with progenitor cells of the cerebellum (9–11). On this basis, genes that are selectively required during development for proliferation and genomic stability of normal neural progenitors may represent attractive targets for MB drug discovery. An interesting group of such genes is represented by primary hereditary microcephaly genes (MCPH), whose loss leads to significant reduction of head circumference and brain volume (12–14). Many MCPH genes have been proposed as specific targets for brain tumors therapy (15–17).

Citron Kinase protein (CITK) is the main product of the *CIT* gene, whose mutations are responsible for MCPH17 (18, 19). CITK is required in neural progenitors for cytokinesis (20, 21), mitotic spindle positioning (22) and chromosomal stability (23). Its loss induces DNA damage and apoptosis in SHH and non-WNT/non-SHH MB cells and reduces growth of both xenograft and transgenic MB (24, 25). CITK downregulation potentiates the effects of ionizing radiations (IR) and cisplatin treatment in reducing growth potential and colony forming activity of MB cells (24). Interestingly, these anti-proliferative effects of CITK loss may be engaged through TP53-dependent and TP53-independent mechanisms (24, 25). Genetic evidence (18), as well as rescue experiments in MB cells (25), indicate that kinase activity is essential for physiological and tumor-sustaining functions.

Although specific inhibitors of CITK activity are not yet available, assessment of 72 inhibitors against 456 human kinases (26) showed that Lestaurtinib has relatively high affinity for this protein.

## 2 Materials and methods

### 2.1 Kinase assays

For radioactive kinase assay, 150 ng of recombinant CITK (ab161903, Abcam, Cambridge, MA, USA) and 500 ng of MYPT1 (654-880) (#12-457, Merck, Sigma-Aldrich, Burlington, MA, USA) were incubated in kinase buffer (50mM Hepes pH7.4, 10mM MgCl2 5mM MnCl2, 0,5mM DTT) with 1μM of ATP and 5 mCi of [γ32P] ATP (6000 Ci mmol-1) (PerkinElmer), for 30 minutes at 30°C.

For ATP-consumption assays, ADP-Glo™ Kinase Assay (V6930, Promega Corporation, Madison, WI, USA) was used according to manufacturer protocol, with the indicated concentrations of Lestaurtinib (Tocris Biotechne group, Minneapolis, MN, USA).

### 2.2 Cell culture

ONS-76 cells (MB subtype: SHH) were kindly provided by Luigi Varesio (Gaslini Hospital, Genoa, Italy) and were cultured in RPMI medium (Euroclone, Milan, Italy) with 10% fetal bovine serum (FBS, Gibco, Gaithersburg, MD, USA). DAOY (MB subtype: SHH) and D283 (MB subtype: non-WNT/non-SHH) cells were obtained from ATCC and were cultured in MEM medium (Euroclone) and 10% FBS (Gibco). D341 cells (MB subtype: non-WNT/non-SHH) were obtained from ATCC and were cultured in MEM medium and 20% FBS (Gibco). Culture media were supplemented by nonessential amino acids, L-glutamine and sodium pyruvate (Gibco). All cell lines, were passed between 5 and 10 times from thawing of the original aliquots and routinely tested for Mycoplasma contamination. All cells were grown at 37°C, in a humidified incubator, with 5% CO_2_.

### 2.3 Transfection of RNAi and constructs

Transfection for RNAi was performed as previously described (24) with published CITK double-stranded RNA (27). Non-targeting pool (Dharmacon, Lafayette, CO) was used as a negative control. Cells plated on six-well plates were transfected using 6.25 μl of the required siRNA (20 μM) together with 1.5 μL Lipofectamine 2000 (Invitrogen, Carlsbad, CA, USA), according to the manufacturer’s instructions. To over express Cherry-tagged wild type CITK and kinase dead constructs, ONS-76 cells were transfected by Transit-it x1 (Mirus Bio LLC, Madison, WI, USA). The constructs used in this manuscript were previously published (27).

### 2.4 Analysis of cell proliferation

To analyze cell proliferation, 25000 ONS-76 and DAOY cells were seeded into 12-well plate, in medium containing DMSO or 100 nM Lestaurtinib and counted in triplicates after 24, 48 and 72 hours with Bio-Rad TC20 Automated Cell Counter. 20000 D283 cells and 30000 D341 cells were seeded into 12-well plate, in medium containing DMSO or 100 nM Lestaurtinib and counted in triplicates after 24, 48, 72 and 96 hours.

### 2.5 Colony forming assay

For colony forming assay, 300 cells were plated at day 1 for ONS-76 and DAOY, in medium containing DMSO or 100 nM Lestaurtinib. For D283 and D341, 6000 and 10000 cells were plated, respectively. Clonogenic assay were stopped after 7-10 days. After medium removal, colonies were incubated for 10 min with Nissl staining and then rinsed in water.

### 2.6 FACS analysis

For cell cycle analysis, cells were plated in 6-well plates and treated with DMSO or 100 nM Lestaurtinib for 24 hours. 50000 cells were collected, centrifuged at 200 g for 5 min, fixed in cold 70% ethanol overnight, washed with PBS two times and finally stained with 5 μL of propidium iodide (from 1 mg/mL of stock) in 500 μL of PBS and RNAse. For apoptosis detection, cells were plated in 6-well plates and treated with DMSO or 100 nM Lestaurtinib for 24 hours. 50000 cells were collected, centrifuged at 200 g for 5 minutes, and resuspended in 300 μL of binding buffer. Cells where then incubated with Annexin V FITC (3 μL) and propidium iodide staining solution (5 μL) for 15 to 30 minutes before flow cytometry.

### 2.7 CellTox™ green cytotoxicity assay

CellTox™ Green Reagent (Promega) was added to each well at the end point of treatment according to the manufacturer’s instructions.

### 2.8 CellTiter-Glo® luminescent cell viability assay

CellTiter-Glo*®* Reagent (Promega) was added to each well at the end point of treatment according to the manufacturer’s instructions.

### 2.9 Antibodies

The following antibodies were used: mouse monoclonal anti-citron (#611377; Transduction Laboratories, BD Biosciences, Franklin Lakes, NJ, USA), mouse monoclonal anti-vinculin (#V9131), mouse monoclonal anti-αTubulin (#T5168) from Sigma-Aldrich; rabbit polyclonal anti-cleaved Caspase 3 (#9661S), anti-TP53 phospho Ser15 (#9284S), anti-γH2AX (S139; 20E3; #2577), anti FLT3 (8F2 #3462) and anti-INCENP (P240, #2807) from Cell Signaling Technology (Danvers, DA, USA); rabbit polyclonal anti-TP73 (#ab14430) and rabbit monoclonal anti-53BP1 (#ab36823) from Abcam (Cambridge, UK); rabbit monoclonal anti-RAD51 (#sc-8349) and rabbit polyclonal anti p21 (#sc-756) from Santa Cruz (Dallas, TX, USA), rabbit polyclonal anti-pTSS INCENP (a kind gift of M.A. Lampson) (28).

### 2.10 Western blotting

Cells and tissues were lysed in RIPA buffer (1% NP40, 150 mmol/L of NaCl, 50 mmol/L of Tris-HCl pH 8, 5 mmol/L of EDTA, 0.01% SDS, 0.005% sodium deoxycholate, Roche protease inhibitors and PMSF) for 10 minutes, at 4 °C. Samples were clarified 10 min at 13,000 rpm at 4 °C. Mouse tissues were homogenized in the same buffer with pellet pestle (Z359971 Sigma-Aldrich). For immunoblots, equal amounts of proteins from both whole-cell lysates were resolved by SDS–PAGE and blotted to nitrocellulose membranes.

### 2.11 Immunofluorescence

Cultured cells were fixed for 10 min at RT with PFA 4%. D341 treated cells were first spun in Thermo Scientific Cytospin 4 (Thermo Fisher Scientific) at 500 rpm, for 10 min, and then processed for immunofluorescence (IF) as previously published (24). Primary antibodies were detected with anti-rabbit Alexa Fluor 488 or 555 and/or anti-mouse Alexa Fluor 488 or 555 (Molecular Probes, Invitrogen), used at 1:1000 dilution, for 30 min. Cells were counterstained with 0.5 µg/mL of DAPI for 10 min and washed with PBS. Finally, cell slides were mounted with Prolong (Thermo Fisher Scientific).

Tumors were fixed in 4% paraformaldehyde in PBS overnight at 4°C, slowly dehydrated with 30% sucrose, embedded with Tissue-TEK (O.C.T., Sakura Finetek, Alphen aan den Rijn, The Netherlands) and frozen by using isopentane. Twenty-micrometers of cryo-sections were rehydrated in PBS and processed for antigen retrieval 1h at 70°C in Antigen Retrieval Buffer (10 mM Na-Citrate pH 6.0, 10% Glycerol in PBS). Primary antibodies were diluted in Tx buffer (0,3% Triton X-100, 0,2% gelatin, 300 mM NaCl in PBS). Secondary antibodies were used at dilution 1:1000 in Tx buffer for 1 hour at RT. TUNEL assay was performed using the Click-iTTM Plus TUNEL Assay (Invitrogen) according to manufacturer protocol. To quantify midbody fluorescence signals, we used Integrated density from Fiji software, that is the sum of the values of the pixels in the image or selection, subtracting the cytoplasmic background; we then calculated the mean of control midbodies and used that value as reference for all midbodies of the same experiment (controls and treated).

### 2.12 Mouse strain

The mouse strain ND2:SmoA1 (expressing the constitutively active point mutant SmoA1 under the Neurod2 promoter in cerebellar granule cells), in congenic C57BL/6J background, was obtained from The Jackson Laboratory.

### 2.13 Experimental animal work

Experiments involving samples from mice treated with DMSO or Lestaurtinib have been performed conforming to the Italian laws on animal experimentation, under permission number 1128/2020-PR, released on 16^th^ November 2020 from Italian Ministry of Health, Department of Public Veterinary Health.

### 2.14 Xenograft assays

Subcutaneous medulloblastoma xenografts were obtained by transplanting in eight-week-old male NOD-SCID mice (Jackson Laboratory) 1x10^6^ ONS-76 cells in the flank. After 4 weeks, mice were treated with 10uL of DMSO or Lestaurtinib 100nM once a week for 4 weeks. Xenograft tumors were measured every week and tumor volume was estimated as 4πr ^3^/3.

### 2.15 Lestaurtinib *in vivo* treatment

Mice showing hunched posture, head tilt or weight loss were anesthetized by intraperitoneal administration of Ketamine (100 mg/ml; MSD Animal Health, Segrate, Italia) supplemented by Xylazine (20 mg/ml; Bayer; Leverkusen, Germany) and then placed in the stereotaxic apparatus. Mice were randomly divided into control and treatment groups. The treatment group received a stereotaxic microinjection of 1 μl of 100 nM Lestaurtinib in PBS in the tumor mass. The control group received a stereotaxic microinjection of 1 μl of DMSO in PBS.

### 2.16 MRI

MRIs were acquired at 7T with a Bruker Neo Avance (Bruker, Ettlingen, Germany) scanner with a 1H quadrature mouse brain volume coil. The animals were scanned every 7 days, starting from 10 weeks of age, to check the presence of tumors through T2-weighted (T2w) high-resolution images acquired with the following parameters: repetition time (TR) =4,000 milliseconds; echo time (TE) = 35.44 milliseconds; rare factor (RF) = 16; slice thickness= 0.5 mm; slice geometry = axial; number of slices = 20;field of view = 2.00 cm;matrix = 256 x 256;number of averages (NAV) = 3; total imaging time: 3 minutes 12 seconds. Animals bearing a tumor mass (size 1–15 mm3) were treated either with a stereotaxic microinjection of 100 nM Lestaurtinib or normal saline (control) starting from week 0 (first administration) to week 4, and imaged by MR with the sequence parameters reported above starting from week 0 to week 7, to monitor tumor progression. Before undergoing MRI, animals were anesthetized by intramuscular injection of 5 mg/kg of xylazine (Rompun; Bayer) and 20 mg/kg of tiletamine/zolazepam (Zoletil 100; Virbac). Tumor volume was assessed in each animal through MR data analysis, carried out with Fiji software. More in details, the tumor area was delineated in each slice of T2w high-resolution images by manually drawing regions of interest along tumor borders. The tumor volume in each slice was estimated multiplying each tumor area for the slice thickness (0.5 mm). Finally, the total tumor volume was estimated by adding up all the single slice volumes.

### 2.17 Survival analysis

Kaplan–Meier method was used to estimate survival. Ten-week-old littermates were divided in DMSO and Lestaurtinib cohorts and treated with once a week for 4 consecutive weeks with a stereotaxic microinjection of DMSO and Lestaurtinib 100 nM, respectively. All mice were euthanized at the onset of symptoms.

### 2.18 Statistical analysis

Statistical analyses were performed using Microsoft Office Excel (Version 16, Microsoft Corporation, Redmond, WA, USA) and GraphPad (Version 8, GraphPad Software, San Diego, CA, USA). Unpaired two-tails Student’s *t*-test was used if not otherwise specified. Data are shown as the mean values of at least 3 independent experiments and standard error of the mean (mean ± SEM). Mann-Whitney test was used to analyze 53BP1 foci and Chi-Square test for percentage distribution using absolute frequency of experiments.

## 3 Results

### 3.1 Lestaurtinib inhibits citron kinase enzymatic functions

**Figure 1:**
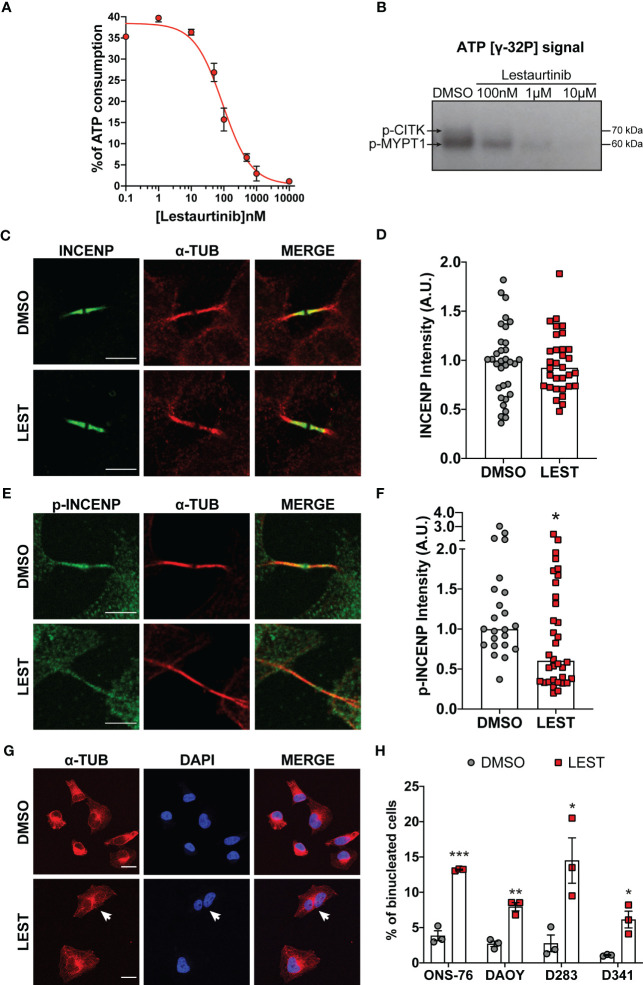
Lestaurtinib inhibits Citron Kinase catalytic activity and induces cytokinesis failure. **(A)** Quantification of ATP consumption was measured with ADP-Glo™ Kinase Assay, by incubating CIT kinase domain with recombinant human MYPT1 (see methods) and the indicated concentrations of Lestaurtinib. **(B)** CITK domain was incubated with MYPT1 in the presence of [γ-32P] ATP. The reactions were then analyzed by SDS-PAGE and autoradiography. p-CITK arrow indicates auto-phosphorylation, p-MYPT1 arrow the substrate phosphorylation. **(C-F)** Analysis of high magnification images of midbodies, in ONS-76 cells treated for 24 hours with DMSO or 100 nM Lestaurtinib (LEST) and immunostained for INCENP **(C)**, phospho(p)-INCENP **(E)**, as well as α-tubulin. Midbody intensity of INCENP and p-INCENP was quantified (**D** and **F**, respectively). Scale bars: 5 μm. **(G)** Representative IF image of ONS-76 cells analyzed 24 hours after treatment with DMSO or 100 nM Lestaurtinib and immunostained for α-tubulin. White arrow indicates a typical binucleated cells. Scale bars: 20 μm **(H)** Quantification of binucleated cells in ONS-76, DAOY, D283 and D341 treated as in **(G)** Each dot indicates an independent biological replicate. >200 cells were counted for each treatment conditions in each experiment. All quantifications were based on at least three independent biological replicates. *P<0.05, **P<0.01 ***P<0.01; unpaired two-tailed Student’s t-test. A.U., arbitrary units.

Since Lestaurtinib could bind CITK with high affinity (26), we evaluated if it could also inhibit CITK catalytic activity. We first resorted to a non-radioactive *in vitro* kinase assay (28) based on the recombinant catalytic domain of the human protein and on MYPT1, a known substrate of myotonic dystrophy kinase family members, which include CITK (29). Increasing concentrations of Lestaurtinib reduced the ATP consumption, with an IC50 of 90 nM (**Figure 1A**). We validated this data using the same enzyme and substrate to perform a radioactive kinase assay. Also in this case, we observed a consistent reduction in both CITK autophosphorylation and MYPT1 phosphorylation, at Lestaurtinib concentrations comparable to those effective in the non-radioactive assay (**Figure 1B**) (29).

We also tested whether, in intact cells, 100 nM Lestaurtinib could affect the phosphorylation of INCENP at residues 834-902, which has been reported as a CITK-dependent event (30). We tested this hypothesis on ONS-76, a MB SHH TP53 wildtype cell line known to be sensitive to CITK depletion. Lestaurtinib treatment significantly reduced phospho-INCENP at midbody, the narrow intracellular bridge that connects two daughter cells at the end of cytokinesis, without altering total protein levels in the same structure (**Figures 1C-F**).

It is well known that loss of CITK leads to cytokinesis failure in many cell types, including MB cells (24, 25). To test whether the same phenotype could be produced by Lestaurtinib, we used 4 different cell lines representing all aggressive MB subtype: ONS-76, DAOY for SHH MB TP53 mutated, D283 and D341 for non-WNT/non-SHH subtypes (7). In all the MB cells tested, 100 nM Lestaurtinib significantly increased the percentage of binucleated cells (**Figures 1G, H**). Time lapse microscopy analysis of Lestaurtinib-treated ONS-76 cells showed that the increase in binucleation is produced because of late cytokinesis failure, following relatively normal cleavage furrow ingression and initial midbody formation (**Figure S1A**, movie. S1 and S2). By western blot, we observed that Lestaurtinib treatment does not alter CITK protein levels in MB cell lines (**Figure S1B, C**). These data indicate that 100 nM Lestaurtinib may significantly inhibit CITK activity, as well as the late stages of cytokinesis which are affected by CITK loss (20, 27).

### 3.2 Lestaurtinib reduces proliferation of MB cells through CITK-sensitive mechanisms and impairs their clonogenic potential

**Figure 2:**
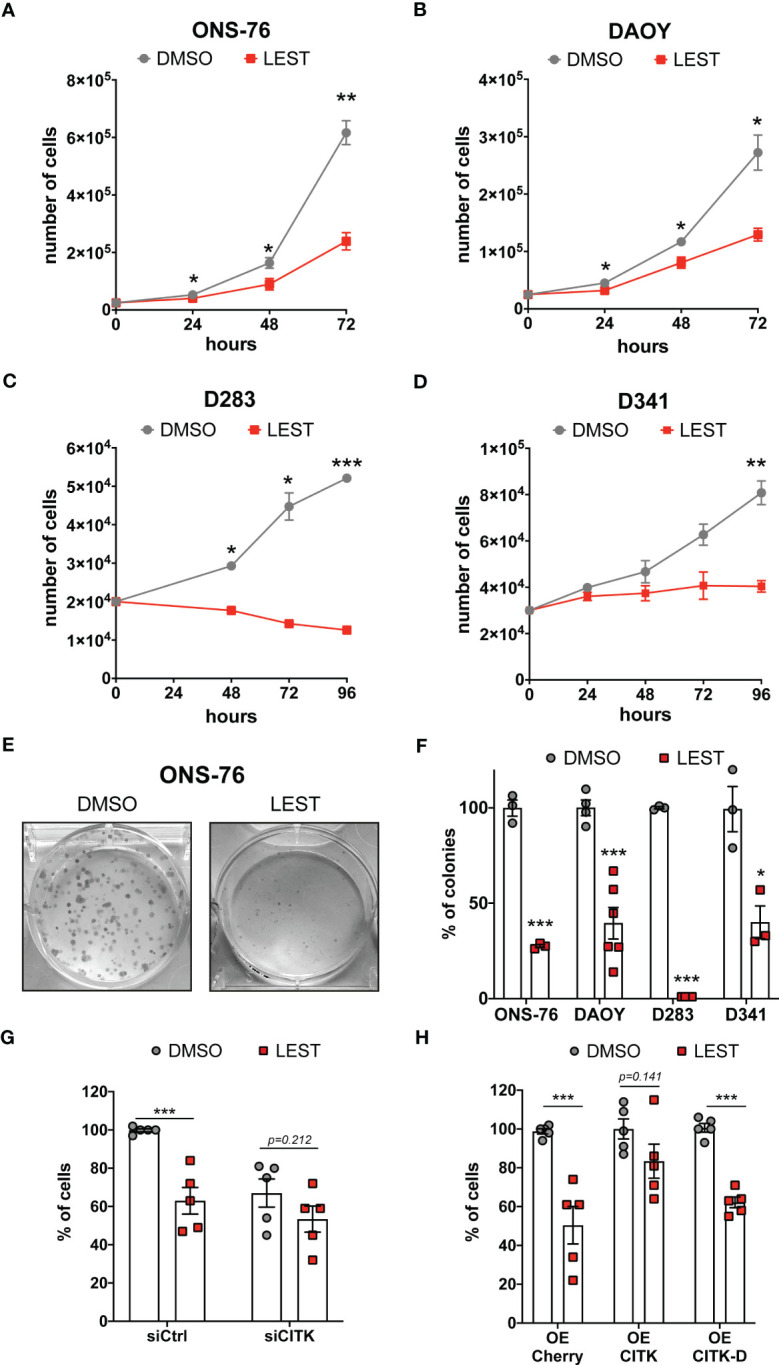
Lestaurtinib reduces MB cells proliferation and impairs the clonogenic potential **(A-D)** Cell proliferation assay: 25,000 ONS-76 **(A)** and DAOY cells **(B)** 20,000 D283 cells **(C)** and 30,000 D341 cells **(D)** were treated with DMSO or 100 nM Lestaurtinib. Growth curves were obtained by assessing cells’ number in each well at the indicated times after treatment. **(E)** Images of ONS-76 colonies 10 days after treatment with DMSO or 100 nM Lestaurtinib and stained with crystal violet. **(F)** Quantification of the percentage of colonies formed after DMSO or 100 nM Lestaurtinib treatment; each dot indicates an independent biological replicate. **(G)** Quantification of ONS-76 cell number 48 hours after transfection of non-targeting (siCtrl) or CITK-specific siRNA (siCITK), treated with DMSO or 100nM of Lestaurtinib during the last 24 hours of the experiment. Each dot indicates the percentage of the corresponding negative control. **(H)** Quantification of ONS-76 cells number 48 hours after transfection of Cherry (control), wild type Cherry-tagged CITK (CITK) and K126A inactive mutant (CITK-D), treated with DMSO or 100nM of Lestaurtinib during the last 24 hours of the experiment. As above, numbers are expressed as percentage of control, in independent biological replicates. All quantifications were based on at least three independent biological replicates. Error bars, SEM. *P<0.05, **P<0.01 ***P<0.01; unpaired two-tailed Student’s t-test.

We next tested whether Lestaurtinib affects the *in vitro* expansion of SHH and non-WNT/non-SHH cell lines. Similarly to CITK downregulation (24, 25), 100 nM Lestaurtinib reduced the proliferation of all the tested MB cell lines (**Figures 2A-D**). Interestingly, the effect was particularly strong in the D283 cell line (**Figure 2C**), which are very sensitive to CITK loss (24). Moreover, Lestaurtinib reduced the clonogenic potential of all tested cell lines, as shown by colony forming assay (**Figures 2E, F**). Both the number and the size of the colonies were reduced.

To assess whether CITK is involved in the anti-proliferative effect produced by Lestaurtinib, we analyzed the growth potential of treated versus untreated ONS-76 cells, expressing control or CITK-specific siRNAs. Compared to the untreated control, cell growth was reduced in all cases, but the effects of Lestaurtinib and CITK loss were not additive (**Figure 2G**). Next, we treated with 100 nM Lestaurtinib ONS-76 cells over expressing Cherry protein (control), as well as Cherry-tagged fusions of wild-type CITK and kinase-dead protein (CITK-D) (31). Interestingly, the active version of CITK rescued the Lestaurtinib anti-proliferative effect, while CITK-D had no effect (**Figure 2H**). Similar results were obtained for DAOY, D283 and D341 cell lines (**Figures S2A, B**). We next assessed the possible role in the MB context of known Lestaurtinib targets. FLT3, the target against which Lestaurtinib was first developed (32, 33), is not expressed at high levels in MB cell lines (**Figure S2C**). The other best known Lestaurtinib target is JAK2 (34). Accordingly, In MB cell lines, phosphorylation of the prominent JAK2 substrate STAT3 was inhibited as well as by the more selective JAK2 inhibitor Ruxolitinib (35) (**Figures S2D, E**). Nevertheless, Ruxolitinib did not impair proliferation of ONS-76 at any tested concentration and significantly reduced DAOY proliferation only at 10 μM concentration (**Figure S2F**). Taken together, these results are consistent with a relatively specific involvement of CITK activity in the growth-inhibitory effects produced by Lestaurtinib on MB cells.

### 3.3 Lestaurtinib induces cell cycle block, apoptosis and TP53 super family activation in MB cells

**Figure 3:**
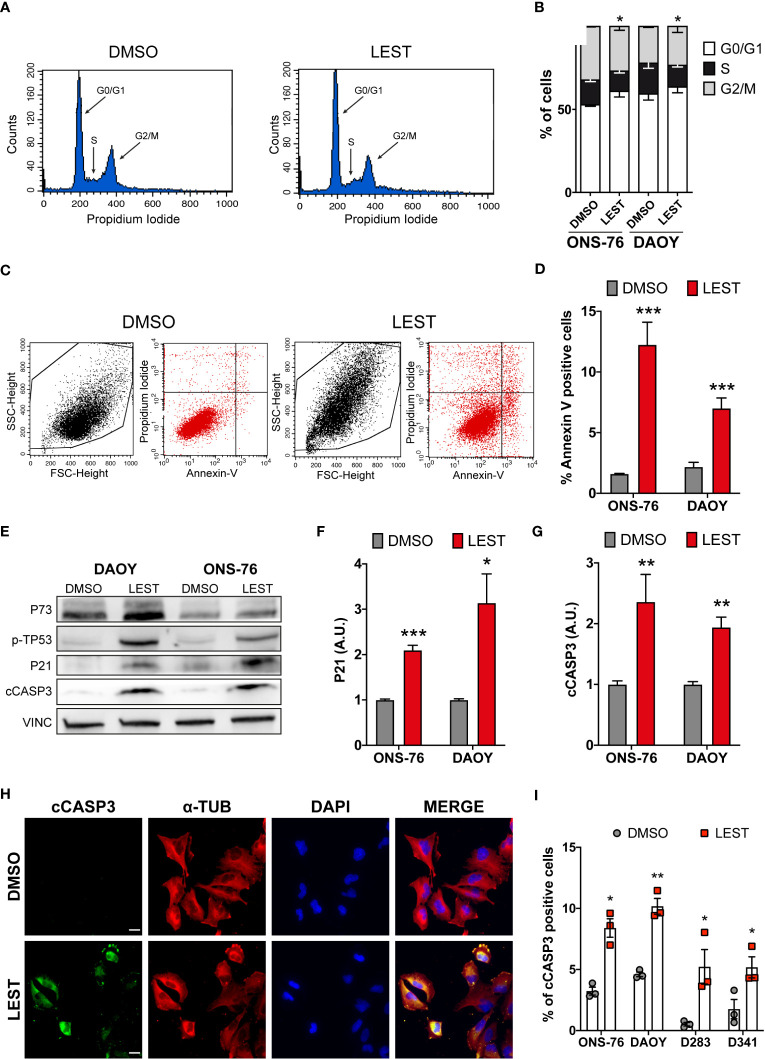
Lestaurtinib induces cell cycle block and apoptosis. **(A)** Exemplar cell-cycle profile of ONS-76 cells treated for 24 hours with DMSO or 100 nM Lestaurtinib. G0/G1, S and G2-M phases are shown. **(B)** Quantification of the percentage of ONS-76 and DAOY treated cells in the different cell-cycle phases; p=0,019 for ONS-76, p=0,04 for DAOY, Chi-Square test. **(C, D)** Quantification of Annexin V positive ONS-76 and DAOY cells, 24 hours after treatment with DMSO or 100 nM Lestaurtinib. **(E)** Western blot analysis of total lysate from ONS-76 cells and DAOY cells, 24 hours after treatment with DMSO or 100 nM Lestaurtinib. The levels of phospho-TP53, TP73, P21 as well as cleaved caspase 3 (cCASP3) were analyzed (loading control vinculin, VINC). **(F, G)** Quantification of the relative density of P21 **(F)**, and cCASP3 **(G)** in 4 replicates of the experiments shown in **(E)** Values are expressed as a ratio over the DMSO control average **(H)** Representative image of ONS-76 cells processed for immunofluorescence 24 hours after treatment with DMSO or 100 nM Lestaurtinib and immunostained for cleaved caspase 3 (cCASP3) and α-tubulin. Scale bars: 5 μm. **(I)** Quantification of the percentage of cCASP3 positive cells in the indicated cell lines, 24 hours after treatment with DMSO or 100 nM Lestaurtinib. All quantifications were based on at least three independent biological replicates. Error bars, SEM. *P<0.05, **P<0.01 ***P<0.01; unpaired two-tailed Student’s t-test. Scale bars: 5 μm. A.U., arbitrary units.

We next assessed whether Lestaurtinib treatment leads to the other prominent phenotypes observed in CITK-depleted MB cells: cell cycle arrest and apoptosis. Flow-cytometry analysis revealed that, in ONS-76 and DAOY, 24 hours of Lestaurtinib treatment produces a significant increase of cells in the G0/G1 phase, as well as a significant reduction of the proportion of tetraploid cells (**Figures 3A, B**). These results are consistent with a strong inhibition of cell-cycle progression, especially if considering the increase of binucleated cells detected by IF (**Figures 1G, H**). Moreover, flow cytometric determination of cells positive for the early apoptosis marker Annexin V revealed that the percentage of ONS-76 and DAOY cells undergoing programmed cell death is significantly increased (**Figures 3C, D**). Consistently, western blot and IF showed increased levels of cleaved caspase 3 (cCASP3) (**Figures 3E-G**). These phenotypes were associated with an induction of phosphorylated-TP53 protein in both ONS-76 and DAOY cells (**Figure 3E**). In addition, the latter cell line, which is known to express a mutated form of TP53 (36), showed increased levels of TP73 protein (**Figure 3E**). Accordingly, in both cell lines Lestaurtinib induced high levels of P21 (**Figures 3E, F**), a prominent downstream effector of TP53 family proteins (25). Similarly, we observed in Lestaurtinib treated D283 and D341 cells an increased percentage of cCASP3 positive cells by IF (**Figures 3H, I**). Finally, we observed in all Lestaurtinib treated cells an increase in cell death (**Figure S3A**), and a reduction of cell viability (**FigureS3B**). These data indicate that, as in the case of CITK knockdown, Lestaurtinib treatment for 24 hours induces cell cycle arrest and cell death in all MB cell lines, possibly involving activation of TP53 family downstream pathways (24, 25).

### 3.4 Lestaurtinib treatment increases DNA damage in MB cells

**Figure 4:**
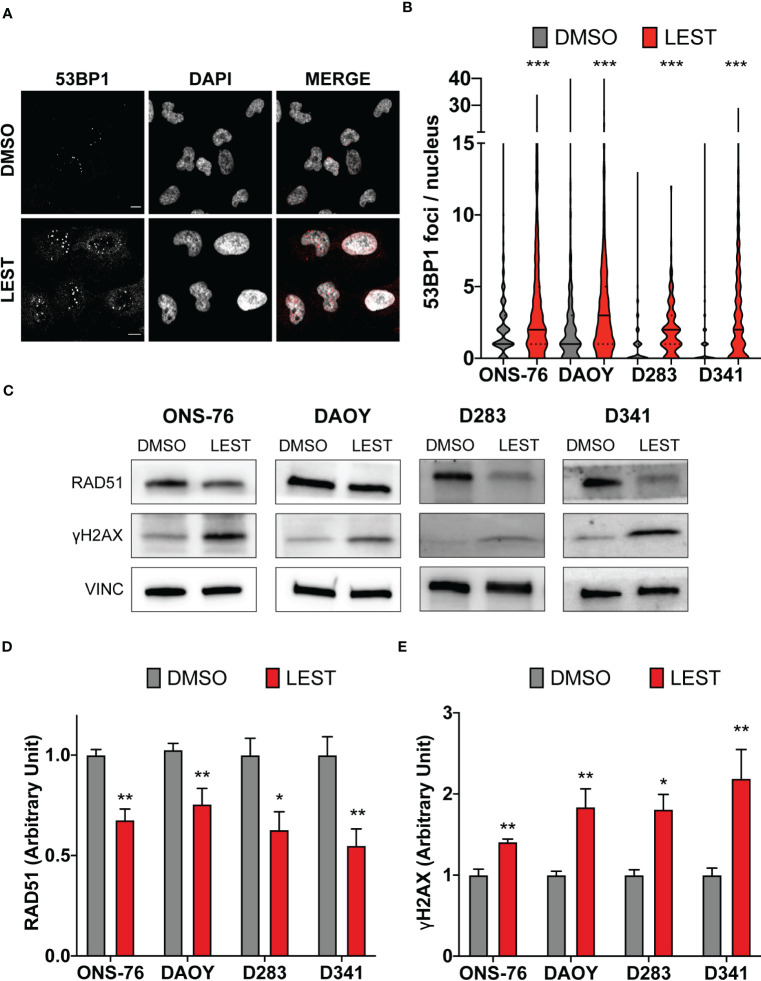
Lestaurtinib induces DNA damage accumulation in MB cells. **(A)** Representative image of ONS-76 cells immunostained for 53BP1 24 hours after treatment with DMSO or 100 nM Lestaurtinib. Scale bars, 10 μm. **(B)** Quantification and 53BP1 nuclear foci in the indicated cell lines, treated as in **(A)**; >250 cells were counted for each treatment conditions in each replicate. **(C)** Western blot analysis of total lysate from the indicated cell lines, 24 hours after treatment with DMSO or 100 nM Lestaurtinib. The levels of RAD51 and γH2AX were analyzed and the internal loading control was vinculin (VINC). **(D, E)** Quantification of the relative density of RAD51 **(D)** and γH2AX **(E)** in the indicated cell lines. All quantifications were based on at least three independent biological replicates. Error bars, SEM. *P<0.05, **P<0.01 ***P<0.01; unpaired two-tailed Student’s t-test for blots, Mann–Whitney U test for 53BP1 foci.

CITK loss leads to DNA double strand breaks accumulation (23–25), associated with reduced nuclear levels of the DNA repair protein RAD51 and impaired homologous recombination (24). Thereby, we wondered whether this also occurs in MB cells after Lestaurtinib treatment. In all cell lines, 24 hours of treatment with 100 nM Lestaurtinib induced significantly increased frequency of 53BP1 foci per nucleus (**Figures 4A, B**), which represent a consolidated marker for DNA double strand breaks. Moreover, compared to control conditions, we observed by western blot increased levels of γ H2AX (marker of any DNA damage), and reduced levels of total RAD51 (**Figures 4C, D**). These data indicate that Lestaurtinib induces DNA damage accumulation in MB cells, suggesting an impairment of homologous recombination.

### 3.5 Lestaurtinib reduces tumor growth in SmoA1 MB bearing mice and increases survival

**Figure 5:**
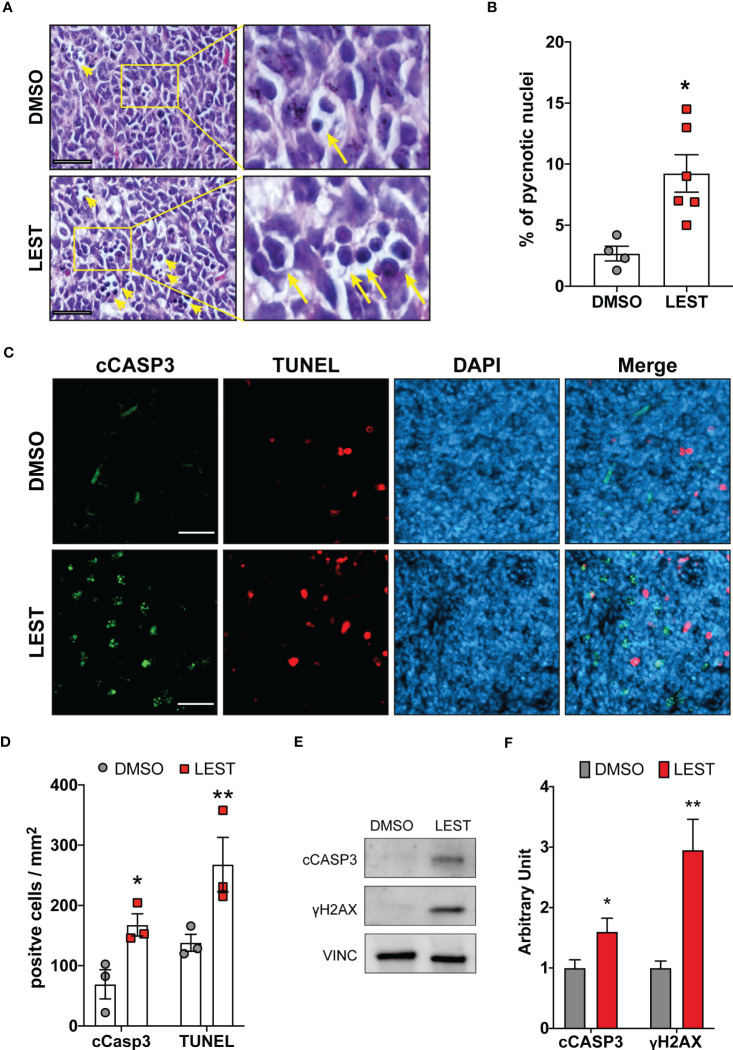
Lestaurtinib induces apoptosis and DNA damage accumulation in vivo. **(A)** Hematoxylin and eosin (H&E) staining of paraffin sections, obtained from MBs that developed in SmoA1 mice, treated with stereotaxic injection of 1μl DMSO or 1μl 100 nM Lestaurtinib (in PBS) for 24 hours. Yellow arrows indicate pyknotic nuclei. Scale bars: 20 μm. **(B)** Percentage of pyknotic nuclei in sections obtained as described in panel **(A)** Each dot indicates a treated animal. **(C)** Frozen sections from tumors obtained as in panel A were labeled by anti-caspase 3 (cCASP3) fluorescent immunostaining and TUNEL assay (red), counterstaining with DAPI. Scale bars: 50 μm. **(D)** Quantification of positive cells for mm^2^ of cCASP3- and TUNEL-positive cells in frozen sections treated as described in panel **(C)** Each dot indicates a treated animal. **(E, F)** Western blot analysis of total cell lysates obtained from tumors obtained as described in **(A)** The levels of cleaved caspase 3 (cCASP3) and γH2AX were analyzed and the internal loading control was GAPDH. Average relative density of cCASP3 and γH2AX in DMSO or Lestaurtinib treated mice (N=6) is reported in panel **(E)** Error bars, SEM. *P<0.05, **P<0.01; unpaired two-tailed Student’s t-test. A.U., arbitrary units.

**Figure 6:**
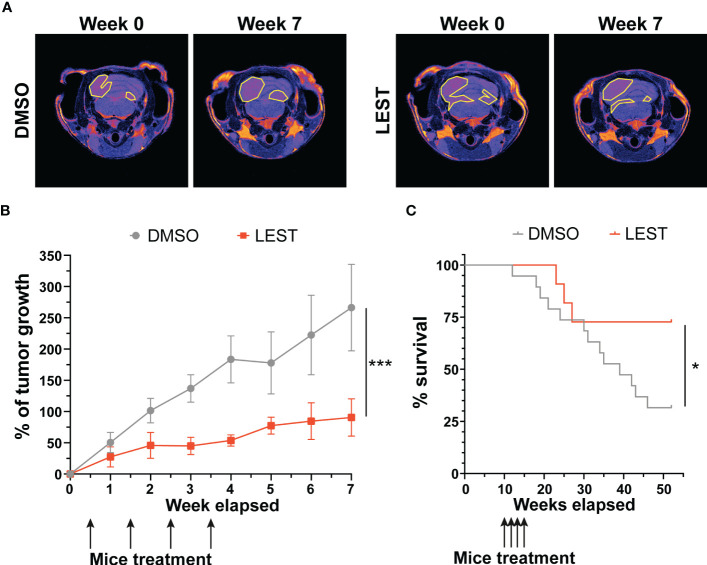
Lestaurtinib inhibits medulloblastoma progression in SmoA1 mice **(A)** Representative T2 weighted MRI-based follow up (from tumor detection, week 0, to last time point monitored, week 7) of mice treated for 4 consecutive weeks with intrathecal injection of DMSO or Lestaurtinib 100nM. Pseudocolorized images are displayed; yellow lines outline the tumor area in MRI sections. **(B)** Quantitative analysis of tumor growth in follow up experiment performed as described in **(A)** Tumor volumes were reconstructed, summing up the corresponding voxels in each section (N = 6). Error bars, SEM. ***P<0.01; different between linear regression of each growth curve. The black arrows indicate Lestaurtinib administration. **(C)**, Kaplan–Meier survival curves of control and mice treated for 4 weeks with Lestaurtinib at 10 weeks of age (N = 19 DMSO, 12 Lest). Log-rank (Mantel–Cox) test was used to compare survival between experimental groups (*P<0,05). The black arrows indicate Lestaurtinib administration.

We next evaluated whether the effects produced by Lestaurtinib *in vitro* may also occur *in vivo*. First, we performed a xenograft assay by subcutaneously injecting into immunodeficient mice ONS-76 cells. Palpable tumors developed within 4 weeks after injection and mice were treated with a weekly injection of 10 uL of DMSO or Lestaurtinib 100nM in the tumor mass. Compared to the rapid growth which we observed in DMSO- treated tumors, Lestaurtinib-injected tumors grew at significantly slower rate (**Figures S4A-B**). To analyze the effects of Lestaurtinib on a model that may more faithfully reproduce the conditions of human MB, we then moved to the ND2:SmoA1 mouse model, in which a constitutively active SmoA1 point mutant is expressed in cerebellar granule cells under the Neurod2 promoter, leading to a high incidence of MB (37). Tumors developing in this model closely mimic the human SHH MB type, since they are generated by accumulation of random mutations after the initial driver event, within immune competent animals (38). In particular we concentrated only on male mice, since the effects of estrogen on MB mouse model is debated (39, 40). To avoid the potential complication of the blood-brain barrier, we delivered Lestaurtinib directly to the tumor mass *via* stereotaxic injections. By histological analysis, we observed a strong increase in the frequency of pyknotic nuclei, throughout the tumor mass of Lestaurtinib treated tumors (**Figures 5A, B**). Accordingly, we detected significantly increased frequency of cells positive for cCASP3 and TUNEL (**Figures 5C, D**) (41–43). Interestingly, the non-affected cerebellar tissue, as well as the rest of CNS, did not show an increased number of apoptotic cells, in both histopathological and immunohistochemical analyses (data not shown). Finally, by western blot, we observed a significant increase of cCASP3 and γ H2AX in the tumor mass of Lestaurtinib treated mice, compared to DMSO treated mice (**Figures 5E, F**). These data indicate that Lestaurtinib injection is effective in inducing DNA damage and apoptosis in an orthotopic model of medulloblastoma. To assess the overall antitumor effect of Lestaurtinib treatment, we monitored by MRI the growth of tumors that received four injections in consecutive weeks. This analysis showed that Lestaurtinib injection impairs medulloblastoma growth, from the beginning of treatment to three weeks after the last injection (**Figures 6A, B**). Finally, we evaluated whether Lestaurtinib significantly ameliorates the survival of SmoA1 mice. In particular, we treated transgenic mice with weekly intratecal Lestaurtinib or control injections, during four consecutive weeks, starting from 10 weeks, a time at which 90% of SmoA1 mice develop MB (25, 38). Long term follow up showed a significant increase in survival of the Lestaurtinib-treated mice (**Figure 6C**). Altogether, these results indicate that Lestaurtinib inhibits SmoA1 medulloblastoma progression.

## 4 Discussion

Protein kinases have emerged as one of the most prominent class of pharmacological targets for cancer treatment. Indeed, 89 kinase inhibitors have been so far approved worldwide (https://www.ppu.mrc.ac.uk/list-clinically-approved-kinase-inhibitors), with main applications in oncology. Although most of these molecules have been raised against specific targets, systematic studies of their kinome interactions revealed that many of them may hit multiple kinases (26, 44). This feature is traditionally considered not very desirable, for the obvious possibility of producing adverse side effects, but it has also been argued that a poly-pharmacological profile is not necessarily a disadvantage for anti-cancer drugs (45–48). Indeed, not only single-target molecules may fail in the long run, due the emergence of drug resistance, but molecules inhibiting several targets may even result in safer and more effective action profiles.

Lestaurtinib (also known as CEP-701) is a Staurosporine derivative that inhibits the FLT3 tyrosine kinase at low nanomolar concentrations. For this reason, it was tested in clinical trials as targeted agent for treatment of acute myeloid leukemia (49) although it didn’t produce significant clinical benefit, as compared to chemotherapy alone (32, 33). Lestaurtinib was also tested in patients with JAK2 mutations and myelofibrosis, who responded to treatment in some cases (34, 50). Considering its effectiveness as a TrkB inhibitor, it has been clinically tested in a Phase I trial in patients with refractory neuroblastoma, who showed sporadic responses (51). All clinical trials indicated that Lestaurtinib is well tolerated. On this ground, and considering that it is a promiscuous inhibitor of many other kinases (26), it is conceivable that Lestaurtinib could be repurposed for treating other tumor types.

In our previous works, we proposed CITK as a promising target for MB treatment, since its loss reduces growth and induces apoptosis of both xenograft and transgenic SHH MBs (24, 25). In the latter case, temporally-controlled genetic deletion of CITK in transgenic MB mice significantly improved survival, without evidence of adverse effects in other organs (25). No specific inhibitors for CITK have been developed so far. We found that Lestaurtinib inhibits CITK catalytic activity with an IC50 of 90 nM (**Figure 1A, B**), a concentration similar to the reported 85 nM K_d_ (26). Moreover, treatment of dividing MB cells at 100 nM significantly alters the midbody abundance of 834-902 phospho-INCENP, which is a known CITK substrate (30) (**Figures 1C-F**). The same concentration of Lestaurtinib produced most of the phenotypic effects obtained in MB cells by CITK depletion. It impairs cell proliferation and clonogenic potential in different types of MB cells (**Figure 2**). The growth inhibition is accompanied by late cytokinesis failure, accumulation of DNA-DSB and apoptosis. Importantly, these effects were also observed after *in vivo* injection, in MB spontaneously arising in the SmoA1 transgenic model. These data significantly extend the current knowledge about the possible biological effects produced by Lestaurtinib, which to our knowledge has not been linked to mitotic alterations or to DNA damage accumulation. Importantly, we obtained evidence that intratecal administration of Lestaurtinib affects the growth of SHH Medulloblastomas spontaneously arising in immunocompetent transgenic mice and increases their long-term survival (**Figure 6**), without producing severe side effects.

Considering the poly-pharmacological phenotype of Lestaurtinib, it is unlikely that all the effects described in this study are due to CITK inhibition. Indeed, the drug can bind with a K_d_ lower than 100 nM to many other kinases, including the well-known mitotic master players CDK2, AURKA and AURKB (26). The latter is a known interactor of CITK during cytokinesis and the cross regulation of the two proteins is crucial to correctly organize midbody proteins (30). Nevertheless, it is conceivable that CITK may play a significant role in the complex networks engaged by Lestaurtinib in MB cells. Indeed, its effects were not additive with CITK depletion and the observed reduction of the proliferative potential was rescued by CITK overexpression (**Figures 2G-H**, **Figure S3A, B**). Under the therapeutic perspective Lestaurtinib could play a beneficial role in MB not only through the novel mechanisms which we have described in this study, but also through inhibition of other kinase-dependent pathways. Considering the critical relevance of mitotic kinases for the expansion of the normal neural progenitors (15)and the promising results obtained in a Phase I trial on refractory neuroblastoma patients (51), the poly-pharmacology profile of Lestaurtinib has the potential of being particularly effective in neuroectodermal tumors, such as MB. In conclusion, based on our results, we propose that Lestaurtinib is a promising lead for MB treatment.

## Data availability statement

The original contributions presented in the study are included in the article/**Supplementary Material**. Further inquiries can be directed to the corresponding author.

## Ethics statement

The animal study was reviewed and approved by Italian Ministry of Health, Department of Public Veterinary Health, Permission number 1128/2020-PR, released on 16th November 2020.

## Author contributions

Conception and design: GP, FD. Development of methodology: GP, RP, FD, ET. Acquisition of data: GI, GP, VB, MG, EF. Analysis and interpretation of data: GI, GP, MG. Writing of the manuscript: GI, GP, FD. All authors contributed to the article and approved the submitted version.
