# Supplementary material for: Lestaurtinib inhibits Citron kinase activity and medulloblastoma growth through induction of DNA damage, apoptosis and cytokinesis failure
Source: Front Oncol. 2023 Jun 19;13:1202585. doi: 10.3389/fonc.2023.1202585 (PMC10315473; doi:10.3389/fonc.2023.1202585)
Supplement: Supplementary file 1 [file DataSheet_1.pdf]

Supplementary Material

Lestaurtinib inhibits Citron kinase activity and medulloblastoma growth through induction of DNA damage, apoptosis and cytokinesis failure.

Gianmarco Pallavicini, Giorgia Iegiani, Roberta Parolisi, Alessia Ferraro, Francesca Garello, Valeria Bitonto, Enzo Terreno, Marta Gai, Ferdinando Di Cunto[*]

*** Correspondence:** Ferdinando Di Cunto: ferdinando.dicunto@unito.it

1 Supplementary Figures

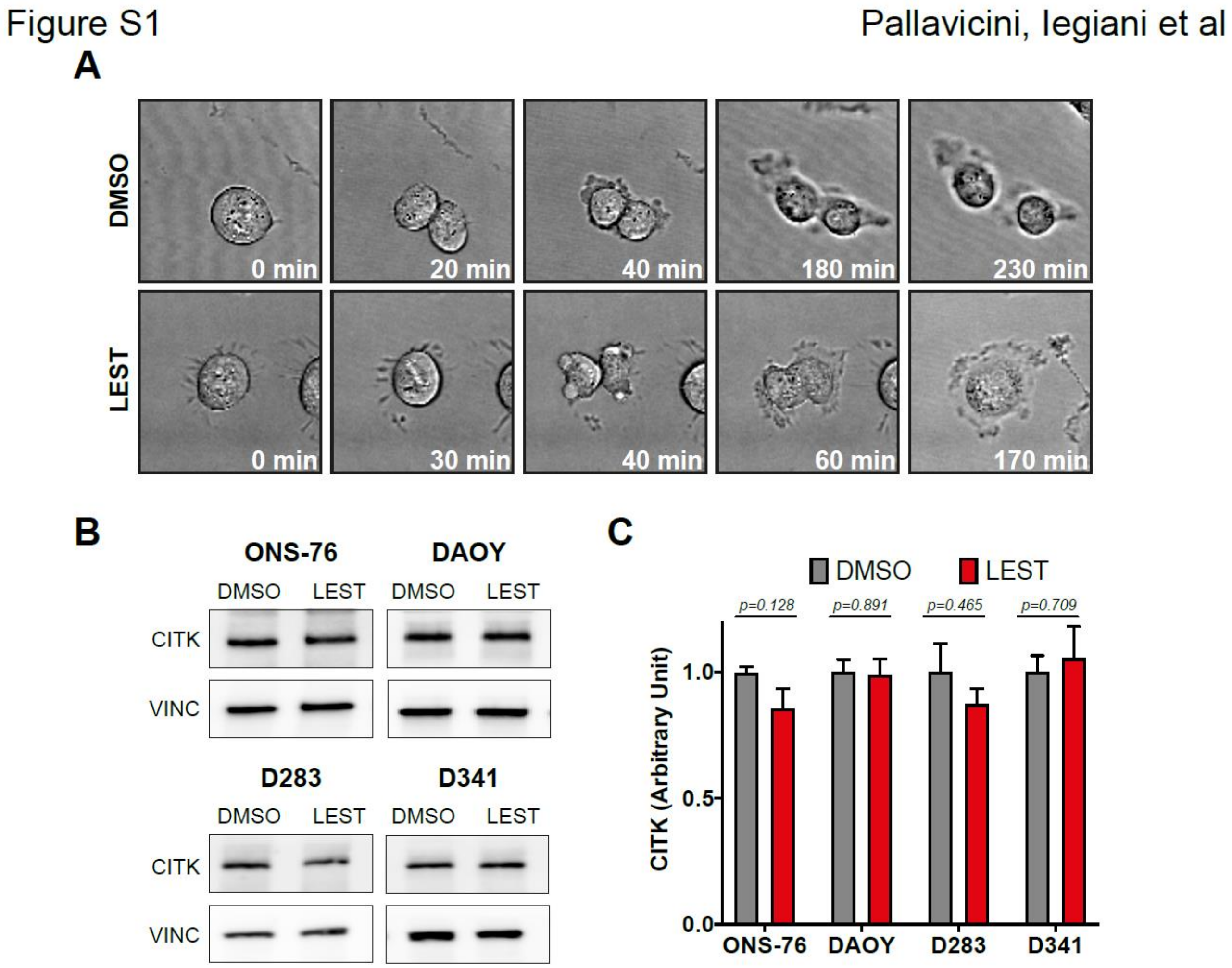

Supplementary Figure 1. Lestaurtinib does not alter CITK protein levels.

(***A***) Representative images of live imaging performed on ONS-76 24 hours after treatment with DMSO or 100 nM Lestaurtinib. Time lapses were recorded overnight with an interval of 5 min. Magnification: 40×
(***B***) Western blot analysis of total lysate from the indicated lines 24 hours after treatment with DMSO or 100 nM Lestaurtinib. The internal control was vinculin (VINC).
(***C***) Quantification of the relative density of CITK in treated cells. All quantifications were based on three independent experiments.

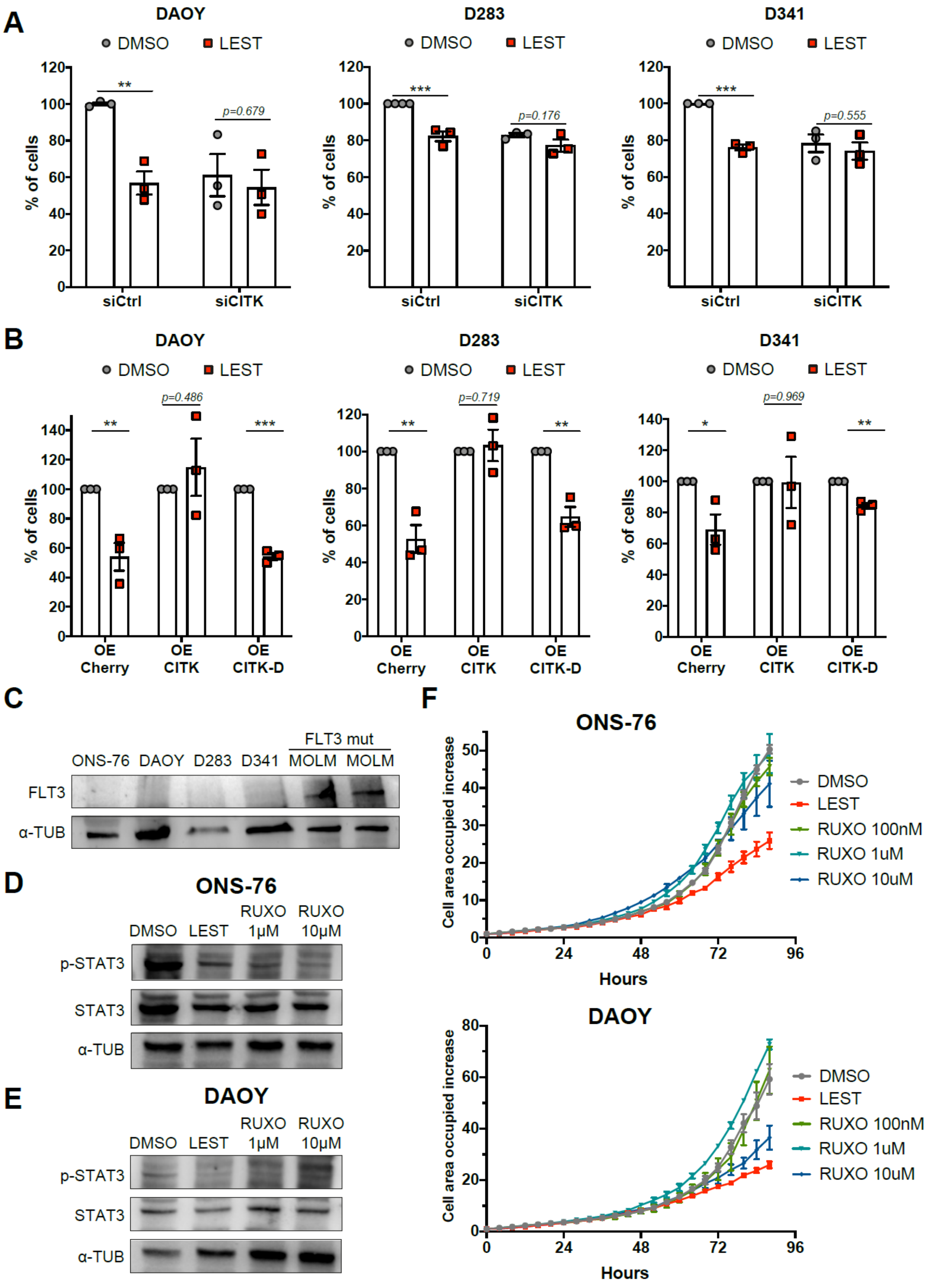
A
DAOY
D283
D341
DMSO
LEST
% of cells
**
***
p=0.679
p=0.176
p=0.555
siCtrl
siCITK
B
p=0.486
p=0.719
p=0.969
*
OE Cherry
OE CITK
OE CITK-D
C
FLT3 mut
ONS-76
DAOY
D283
D341
MOLM
MOLM
FLT3
α-TUB
D
ONS-76
DMSO
LEST
RUXO 1μM
RUXO 10μM
p-STAT3
STAT3
α-TUB
E
DAOY
F
ONS-76
Cell area occupied increase
Hours
DMSO
LEST
RUXO 100nM
RUXO 1uM
RUXO 10uM
DAOY

Supplementary Figure 2. Lestaurtinib effects occur through CITK-sensitive mechanisms.

(***A***) Quantification of the percentage of DAOY, D283, D341 cells obtained 48 hours after transfection of non-targeting (siCtrl) or CITK-specific siRNA (siCITK), treated with DMSO or 100nM of Lestaurtinib during the last 24 hours of the experiment. Each dot indicates an independent biological replicate, referred to the corresponding control.
(***B***) Quantification of the percentage of DAOY, D283, D341 cells obtained 48 hours after transfection of Cherry (control), wild type Cherry-tagged CITK (CITK) and K126A inactive mutant (CITK-D), treated with DMSO or 100nM of Lestaurtinib during the last 24 hours of the experiment. Each dot indicates an independent biological replicate, referred to the corresponding control.
(***C***) Western blot analysis of total lysate from ONS-76, DAOY, D283 and D341 compared to MOLM cells. The levels FLT3 were analyzed (loading control tubulin, TUB).
(***D-E***) Western blot analysis of total lysate from ONS-76 and DAOY cells, 24 hours after treatment with DMSO or 100 nM Lestaurtinib or 1μM and 10μM Ruxolitinib (RUXO). The levels of phospho-STAT3, STAT3 were analyzed (loading control tubulin, TUB).
(***F***) Cell proliferation assay of ONS-76 and DAOY; cells were treated with DMSO or 100 nM Lestaurtinib or 100nM, 1μM and 10μM Ruxolitinib (RUXO). Growth curves were obtained by assessing cells' area in each well at the indicated times after treatment.
All quantifications were based on at least three independent biological replicates. Error bars, SEM. *P<0.05, **P<0.01 ***P<0.01; unpaired two-tailed Student's t-test.

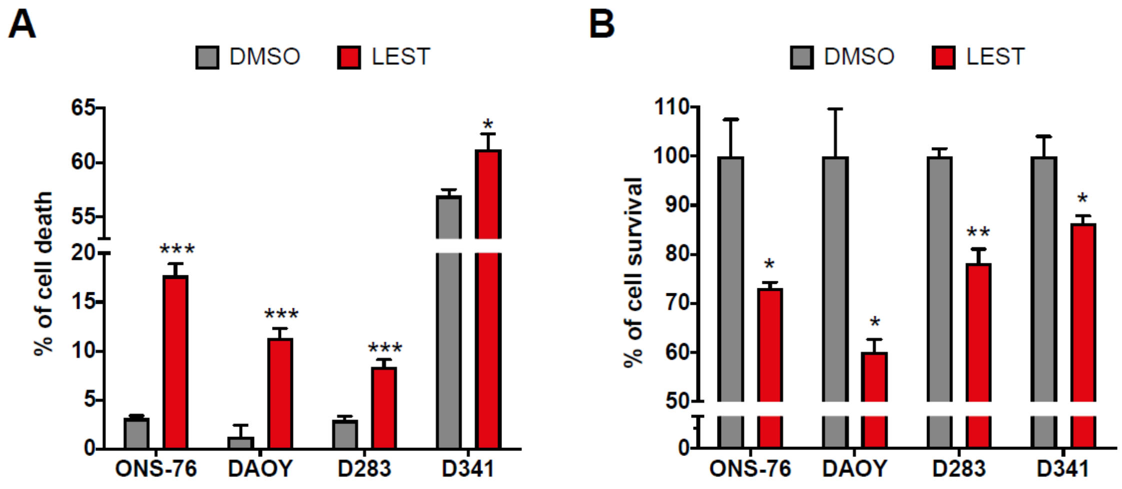

Supplementary Figure 3. Lestaurtinib induces cell death in MB cell lines.

(***A***) Cell death was measured in the indicated lines by CellToxTM Green Cytotoxicity assay, 24 hours after treatment with DMSO or 100 nM Lestaurtinib.
(***B***) Cell viability was measured in the indicated lines by CellTiter-Glo® Luminescent Cell Viability Assay, 24 hours after treatment with DMSO or 100 nM Lestaurtinib.
All quantifications were based on three independent biological replicates. Error bars, SEM. *P<0.05, **P<0.01 ***P<0.01; unpaired two-tailed Student's t-test. Chi2 for percentage distribution using absolute frequency of experiments.

Figure S4 Pallavicini, Iegiani et al

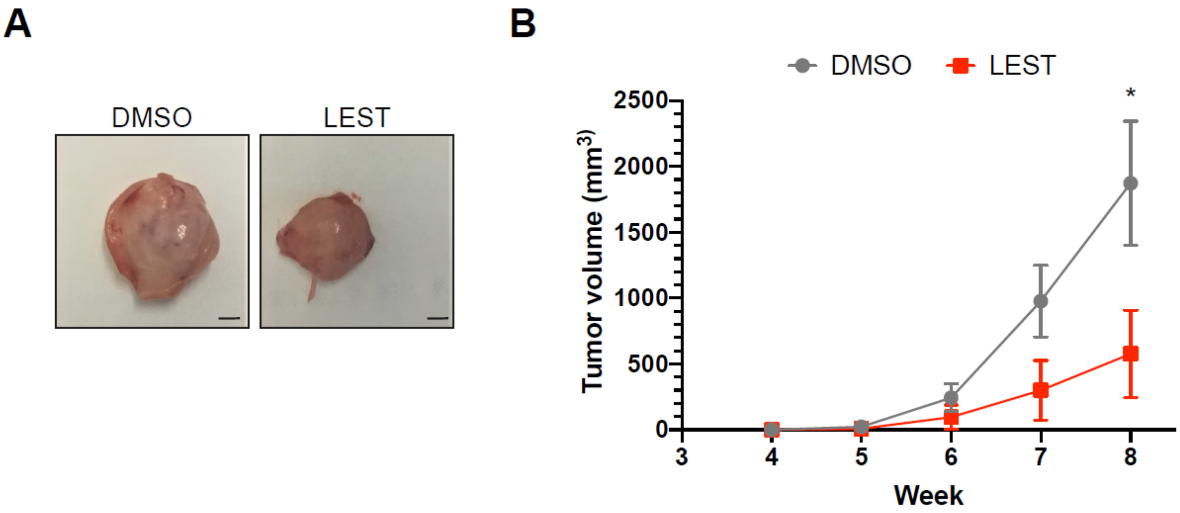

Supplementary Figure 4. Lestaurtinib decreases the growth of xenograft tumors.

(***A***) Representative pictures of xenograft tumors, obtained by subcutaneous injection of ONS-76 cells in NOD-SCID mice. Lestaurtinib treatment was started 4 weeks after cells injection when tumors became palpable, and tumor growth was monitored for 4 additional weeks. DMSO or Lestaurtinib 100 nM was injected in the tumor mass.
(***B***) Analysis of xenograft tumors growth, performed by measuring subcutaneous tumor diameter. All quantifications were based on 7 independent biological replicates. Error bars, SEM. *$P<0.05$, unpaired two-tailed Student’s t-test.
